# MicroRNA-145 inhibits the proliferation, migration and invasion of the human TCA8113 oral cancer line

**DOI:** 10.3892/ol.2013.1621

**Published:** 2013-10-11

**Authors:** YUAN SHAO, SHAO-QIANG ZHANG, FANG QUAN, PENG-FEI ZHANG, SHENG-LI WU

**Affiliations:** 1 Department of Otorhinolaryngology, The First Affiliated Hospital of Xi’an Jiaotong University School of Medicine, Xi’an, Shaanxi 710061, P.R. China; 2 Department of Hepatobiliary Surgery, The First Affiliated Hospital of Xi’an Jiaotong University School of Medicine, Xi’an, Shaanxi 710061, P.R. China

**Keywords:** oral cancer cell, microRNA-145, proliferation, migration, invasion

## Abstract

The aim of this study was to investigate the effect of microRNA (miR)-145 on the proliferation, migration and invasion of the human oral cancer line, TCA8113. Expression levels of miR-145 in TCA8113 cells were detected by quantitative PCR. miR-145 was transfected into human TCA8113 oral cancer cells and the proliferation, migration and invasion abilities of treated TCA8113 cells were detected by proliferation, migration and invasion assays, respectively. The expression levels of miR-145 in TCA8113 cells were significantly lower than those in human normal oral keratinocytes (P<0.05). Cellular proliferation, migration and invasion abilities in the miR-145 transfection group were significantly lower than those in the control group (all P<0.05). High miR-145 expression was found to negatively regulate the proliferation, migration and invasion of TCA8113 cells. Results of the present study indicate that the expression of miR-145 may be associated with the genesis and development of human oral cancer.

## Introduction

Oral cancer is the sixth most common type of cancer worldwide. The annual estimated incidence is ~275,000 (1) and has recently been reported to be increasing in frequency (2). Despite advances in diagnostic techniques and improvement in treatment modalities, the prognosis of oral cancer remains poor, mainly owing to the high rate of local and regional recurrence and to the development of new malignant changes within the original field of precancerization (3–5). Numerous pathways have been reported to be activated during oral cancer progression (6). In order to develop therapeutic approaches that hit multiple targets, the identification of molecular targets for oral cancer is urgently required.

MicroRNAs (miRNAs) are short single-stranded nucleotide RNA molecules, which function as master regulators of gene expression by post-transcriptional modifications of target mRNAs (7). These noncoding RNAs are emerging as important modulators in cellular pathways and appear to play a key role in tumorigenesis (8–10). With increasing understanding of the cellular behaviors affected by them, modulation of miRNA activity may provide exciting opportunities for cancer therapy.

miRNAs have been reported to serve as tumor suppressors (11). Specifically, it has been indicated that miR-145 is a tumor suppressor capable of inhibiting tumor cell growth (12) and expression levels of miR-145 have been found to be decreased in human lung adenocarcinoma (13). However, the role of miR-145 in human oral cancer remains largely unknown. In the present study, the expression levels of miR-145 were investigated in the human TCA8113 oral cancer line and the effect of miR-145 transfection on the proliferation, migration and invasion abilities of TCA8113 cells was determined.

## Materials and methods

### Cell lines

Human oral TCA8113 cancer and human normal oral keratinocytes (hNOK) cell lines were obtained from the Center of Biomedical Experimental Research at the Xi’an Jiaotong University School of Medicine (Xi’an, China).

### Cell culture

TCA8113 and hNOK cells were separately cultured in Dulbecco’s modified Eagle’s medium (Invitrogen Life Technologies, Carlsbad, CA, USA) containing 10% fetal bovine serum (FBS) and 1% penicillin/streptomycin (Invitrogen Life Technologies), and incubated at 37°C in an atmosphere containing 5% CO_2_.

### Quantitative PCR (qPCR) of miR-145

qPCR was used to measure miR-145 expression levels in hNOK and TCA8113 cells. miR-145 was amplified using the Bulge-Loop™ miRNA qRT-PCR Primer Set (Guangzhou RiboBio Co., Ltd., Guangzhou, China) (14). The thermal profile for the qPCR was 95°C for 1 min, followed by 40 cycles of 95°C for 10 sec, 60°C for 20 sec and 72°C for 5 sec on a Bio-Rad CFX96 RT-qPCR system (Bio-Rad, Hercules, CA, USA). All qPCR, including no-template controls, were performed in triplicate. Expression levels of miR-145 were evaluated using the comparative threshold cycle method and normalized against U6.

### TCA8113 cell transfection with miR-145

The miR-145 expression vector (miRNASelect pEP-miR-145) and miRNA negative control vector (miRNASelect pEP-miR-Null) were obtained from Cell Biolabs Inc., (San Diego, CA, USA). TCA8113 cells were transfected with pEPmiR-145 or pEP-miR-Null using Effectene transfection reagent (Qiagen, Hilden, Germany) according to the manufacturer’s instructions. Vector DNA (1 μg) was diluted in 100 μl extracellular buffer mixed with 8 μl enhancer and incubated for 2 min. Effectene transfection reagent (5 μl) was added to the DNA-enhancer mixture and incubated for 10 min to allow transfection complex formation. The normal growth medium was replaced by 2.5 ml antibiotic free medium containing 10% (v/v) FBS during complex formation. The transfection complex was then applied to the cells and incubated for 48 h. Transfection efficiency was analyzed by qPCR at 48 h following transfection, as aforementioned.

The following four groups were established in this study: blank control, reagent (cells treated with transfection reagent), negative control (cells treated with transfection reagent plus pEP-miR-Null) and miR-145 group (cells treated with transfection reagent plus pEPmiR-145).

### Cell proliferation assay

The 3-(4,5-dimethylthiazol-2-yl)-2,5-diphenyltetrazolium bromide (MTT; Sigma-Aldrich, St. Louis, MO, USA) colorimetric assay was used to screen for cell proliferation. In brief, cells were seeded into eight 96-well plates at a density of 2×10^3^ cells/well and maintained in normal growth medium in a 5% CO_2_ humidified incubator at 37°C for 48, 72 and 96 h following treatment. Subsequently, 20 μl MTT (5 mg/ml) was added into each well and cell culture was continued for a further 4 h. Following aspiration of the medium, the cells were lysed with dimethylsulfoxide (Sigma-Aldrich). The absorbance was measured using a microplate reader at 490 nm. The cell growth curve was plotted with optical density values as ordinate against time as abscissa. The experiment was repeated three times.

### Cell migration assay

The effect of miR-145 transfection on TCA8113 cell migration was measured as the ability of cells to migrate through Transwell filters (6.5-mm diameter; 5-mm pore size). Transwell filters were coated with reconstituted basement membrane substance (Matrigel; BD Biosciences, San Diego, CA, USA) for 1.5 h prior to adding the cells. At 24 h following miR-145 transfection, cells were detached by trypsinization and 1×10^5^ cells were seeded into Transwell filters in 100 ml starvation medium. Subsequently, 500 ml growth medium was placed in the lower compartment and the cells were left to migrate for 24 h. Nonmigrated cells were removed using a cotton swab and the transmigrated cells at the rear side of the filter were stained with Giemsa. TCA8113 cells on each filter were counted at magnification, ×400 to quantitate TCA8113 cell migration. Images of three random fields from three replicate wells were captured. Migration was determined as the mean of cells that had migrated per ×400 field and expressed as a percentage of the blank control.

### In vitro invasion assay

TCA81135 cell invasion was evaluated using 24-well Transwell units with 8-μm porosity polycarbonate filters. The filters were coated with 50 μl Matrigel (8 mg/ml). The coated filters were air-dried at 4°C prior to the addition of the cells. The basement membrane was hydrated with 50 μl serum-free RPMI-1640 medium 30 min prior to use. At 24 h following miR-145 transfection, cells were digested with trypsin and the cell density was adjusted to 1×10^6^ cells/ml using serum-free RPMI-1640 medium. A total of 200 μl cell suspension was added into each upper Transwell chamber and 600 μl RPMI-1640 medium containing 5% fetal bovine serum was added into the lower chamber. There were three duplicates for each cell group. Following this, the cells were incubated for 24 h in a humidified atmosphere of 5% CO_2_ at 37°C. Cells were fixed with methanol and stained with Giemsa. Cells on the upper surface of the filter were removed by wiping with a cotton swab and invasion was determined by counting the cells that migrated to the lower side of the filter with optical microscopy at magnification, ×400. A total of five visual fields at the center and in the surrounding areas were counted, and the average was calculated (15). The experiment was repeated three times.

### Statistical analysis

All data are presented as the means ± SEM. Statistical analysis was performed using SPSS 16.0 software (SPSS, Inc., Chicago, IL, USA). Differences among groups were tested by one-way analysis of variance. P<0.05 was considered to indicate a statistically significant difference.

## Results

### Expression of miR-145 in hNOK and TCA8113 cells

As demonstrated by qPCR, the expression levels of miR-145 in human oral cancer TCA8113 cells were ~50% of those of the hNOK cells (0.342±0.029 vs. 0.695±0.033). There was a significant difference between the two groups (P<0.05).

### Validation of miR-145 transfection in TCA8113 cells

**Figure 1:**
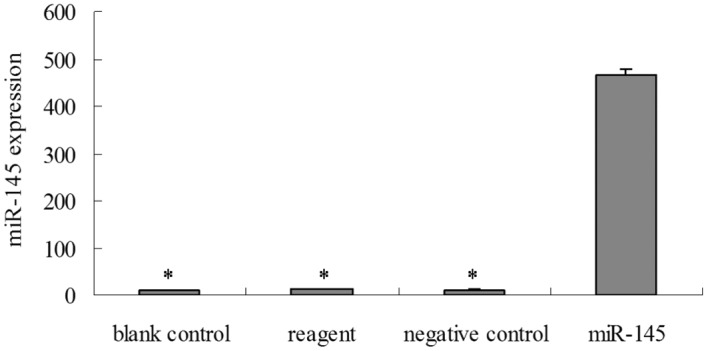
miR-145 expression levels in TCA8113 cells. TCA8113 cells were transfected with pEPmiR-145 for 48 h and then collected for qPCR. The expression of miR-145 was significantly increased in TCA8113 cells. ^*^P<0.05, vs. blank control, reagent and negative control group cells. miR, microRNA; qPCR, quantitative PCR.

qPCR was performed to validate the transfection of miR-145 in TCA8113 cells. As shown in Fig. 1, compared with the blank control, reagent and negative control group cells, the expression of miR-145 in the miR-145 group cells was significantly increased at 48 h following transfection (P<0.05), which continued for at least 96 h (data not shown).

### Transfection with miR-145 decreases cell proliferation in TCA8113 cells

**Figure 2:**
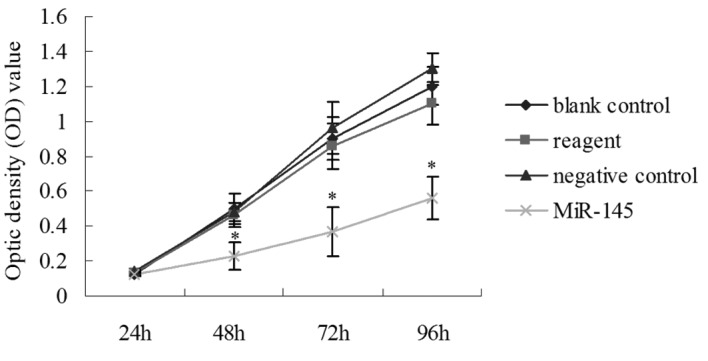
Transfection with miR-145 decreases cell proliferation in TCA8113 cells. TCA8113 cell proliferation was analyzed using the MTT assay. TCA8113 cells were monitored for 96 h and the average OD_490_ (± SD) for each cell group is shown. TCA8113 cells transfected with pEPmiR-145 showed reduced cell growth relative to the blank control, reagent and negative control group cells at 48, 72 and 96 h following treatment, respectively (^*^P<0.05, vs. blank control, reagent and negative control group cells). miR, microRNA; MTT, 3-(4,5-dimethylthiazol-2-yl)-2,5-diphenyltetrazolium bromide.

To examine whether miR-145 transfection had an effect on TCA8113 cell growth, an MTT cell proliferation assay was performed. Compared with the blank control, reagent and negative control group cells, the miR-145 group cells showed decreased cell proliferation at 48, 72 and 96 h post-transfection, which is consistent with the role of miR-145 in cell growth in TCA8113 cells (P<0.05; Fig. 2).

### Transfection with miR-145 reduces cell migration in TCA8113 cells

**Figure 3:**
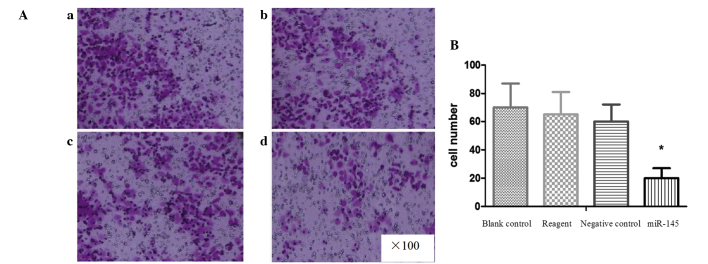
Transfection of miR-145 reduces cell migration in TCA8113 cells. (A) A Matrigel Transwell invasion assay was used to test the migration ability of TCA8113 cells (from various groups) to pass through the basement membrane. (a) Blank control, (b) reagent, (c) negative control and (d) miR-145 group cells. (B) Cell number (mean ± SD) per visible field (^*^P<0.05, vs. blank control, reagent and negative control group cells). miR, microRNA.

The effect of miR-145 transfection on the cell migration ability of TCA8113 cells was investigated by Transwell invasion assay (Fig. 3A). The results indicated that miR-145-transfected cells had a significantly reduced ability to pass through the basement membrane when compared with the cells in the other three groups (all P<0.05; Fig. 3B). These data are consistent with the hypothesis that miR-145 may serve as a tumor suppressor.

### Transfection with miR-145 reduces the invasive ability of TCA8113 cells

**Figure 4:**
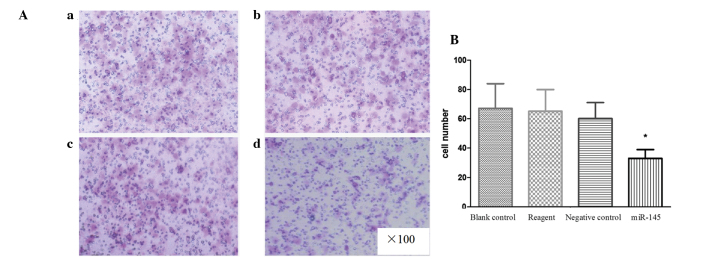
Transfection of miR-145 reduces the invasive ability of TCA8113 cells. (A) A Matrigel Transwell invasion assay was used to test the ability of TCA8113 cells (from various groups) to invade the filter membrane (a) Blank control, (b) reagent, (c) negative control and (d) miR-145 group cells. (B) Cell number (mean ± SD) per visible field (^*^P<0.05, vs. with blank control, reagent and negative control group cells). miR, microRNA.

The invasive ability of TCA8113 cells was investigated using a Matrigel invasion assay. The invasion assay results indicated that miR-145-transfected TCA8113 cells had a significantly lower ability to pass through the basement membrane compared with the cells in the other three groups (all P<0.05; Fig. 4A and B). These result indicate that miR-145 is involved in the suppression of TCA8113 cell invasion.

## Discussion

At present, changes in the miRNA profile in various cancer cells and their roles in carcinogenesis have been increasingly analyzed (16). Studies have shown that miR-145 is closely associated with tumorigenesis and the expression levels of miR-145 have been found to be significantly downregulated in bladder (17), breast (18), colorectal (19), esophageal (20) and gastric cancers (21). Downregulated expression levels of miR-145 in oral cancer have been shown previously in a hamster oral squamous cell cancer model (22). However, the role of miR-145 in human oral cancer tumorigenesis remains poorly understood. In the present study, the expression of miR-145 was investigated in the human TCA8113 oral cancer line, and the effect of miR-145 transfection on the proliferation, migration and invasion abilities of TCA8113 cells was also observed.

qPCR analysis indicated that miR-145 expression levels were significantly lower in TCA8113 cells compared with hNOK cells, indicating that miR-145 may be associated with the genesis of human oral cancer. These data are consistent with previous studies showing decreased miR-145 expression in a variety of malignant tumors. Sachdeva *et al* showed that the expression levels of miR-145 decreased gradually during the transition from normal breast tissue to cancer tissue (23). Chen *et al* also reported that the expression levels of miR-145 declined gradually from the tumorigenesis to the progression stage in prostate cancer (24). In addition, a previous study by Drebber *et al* showed that, in patients with advanced colon cancer undergoing neoadjuvant chemoradiotherapy, a significant upregulation of miR-145 in post-therapeutic tumor tissue was noted compared with that in pre-therapeutic tumor tissue. Patients with low intratumoral post-therapeutic expression had a significantly poorer response to neoadjuvant therapy compared with patients with a high expression of miR-145 (25). These results indicate that miR-145 may be an important molecular biomarker in early diagnosis and prediction of treatment response and prognosis of tumors.

At present, the diagnosis and clinical staging of tumors are mainly based on conventional histology and radiological imaging. Due to its convenience and non-invasiveness, blood sampling for the detection of miRNAs as tumor markers has been increasingly applied in recent years. Serum miR-132, miR-26a, let-7b and miR-145 have been reported as potential candidates for novel biomarkers in serous ovarian cancer (26). Due to its decreased expression in TCA8113 cells, we hypothesize that miR-145 may be used as a potential biomarker in the early diagnosis of oral cancer.

We further investigated the effect of miR-145 transfection on *in vitro* proliferation, migration and invasion of TCA8113 cells. Upregulation of miR-145 resulted in a suppression of tumor cell proliferation, migration and invasion. Our observations are consistent with previous studies on the functional roles of miR-145. Shi *et al*(27) reported that, in colon cancer cells, miR-145 is directly bound to the insulin receptor substrate-1 (IRS-1) 3′-untranslated region and downregulates IRS-1 protein, inhibiting the growth of colon cancer cells. Gregersen *et al*(19) employed a microarray-based approach to identify miR-145 targets in colon cancer cells, and YES and STAT1 were verified as direct miR-145 targets. In the PC3 prostate cancer cell line, Zaman *et al*(28) found that overexpression of miR-145 by transfection resulted in increased apoptosis and an increase in cells in the G2/M phase. Microarray analysis of miR-145-overexpressing PC3 cells showed upregulation of the pro-apoptotic gene, TNFSF10. In HCT-116 and MCF-7 cells, Sachdeva *et al*(23) showed that c-Myc is a direct target of miR-145. In addition, the blockade of miR-145 by anti-miR-145 was able to reverse p53-mediated c-Myc repression, defining a role for miR-145 in the post-transcriptional regulation of c-Myc by p53 and indicating that miR-145 provides a direct link between p53 and c-Myc in this gene regulatory network. miR-145 was also found to play a negative regulatory role in cell growth through RTKN (29), OCT, SOX-2 and KLF4 pathways (30). A further study (12) showed that miR-145 significantly suppresses cell invasion in MCF-7 and HCT-116 cells, and that miR-145 is also able to suppress lung metastasis in an experimental metastasis animal model. This miR-145-mediated suppression of cell invasion is, in part, due to the silencing of the metastasis gene, mucin 1 (MUC1). Furthermore, suppression of MUC1 by miR-145 causes a reduction of β-catenin as well as oncogenic cadherin 11. Overall, these results indicate that, as a tumor suppressor, miR-145 inhibits not only tumor growth but also cell invasion and metastasis, and miR-145 is a promising new therapeutic target for the treatment of various types of cancer, including oral cancer. In the future, studies of the regulation of target genes by miR-145 in oral cancer TCA8113 cells are likely to continue to identify the action mechanisms of miR-145, which may be significant for the diagnosis and treatment of oral cancer through miR-145.

In conclusion, our data indicate that the expression levels of miR-145 in TCA8113 cells are significantly lower than those of hNOK cells. miR-145 may be a valuable molecular biomarker for the early diagnosis of oral cancer. Cellular proliferation, migration and invasion abilities in miR-145-transfected TCA8113 cells were significantly decreased, indicating that miR-145 may participate in oral cancer genesis and progression. Thus, miR-145 is a potential therapeutic target for the treatment of oral cancer.
